# Efficacy of Sitafloxacin for *Mycoplasma genitalium* in an Era of Increasing Antimicrobial Resistance

**DOI:** 10.1093/ofid/ofad590

**Published:** 2023-11-22

**Authors:** Ranjit S Samra, Erica L Plummer, Lenka A Vodstrcil, Ivette Aguirre, Emily J Clarke, Christopher K Fairley, Eric P F Chow, Catriona S Bradshaw

**Affiliations:** Melbourne Sexual Health Centre, Alfred Health, Carlton, Victoria, Australia; Department of Infectious Diseases, Alfred Hospital, Alfred Health, Melbourne, Victoria, Australia; Melbourne Sexual Health Centre, Alfred Health, Carlton, Victoria, Australia; Central Clinical School, Monash University, Melbourne, Victoria, Australia; Melbourne Sexual Health Centre, Alfred Health, Carlton, Victoria, Australia; Central Clinical School, Monash University, Melbourne, Victoria, Australia; Centre for Epidemiology and Biostatistics, Melbourne School of Population and Global Health, University of Melbourne, Melbourne, Victoria, Australia; Melbourne Sexual Health Centre, Alfred Health, Carlton, Victoria, Australia; Melbourne Sexual Health Centre, Alfred Health, Carlton, Victoria, Australia; Melbourne Sexual Health Centre, Alfred Health, Carlton, Victoria, Australia; Central Clinical School, Monash University, Melbourne, Victoria, Australia; Melbourne Sexual Health Centre, Alfred Health, Carlton, Victoria, Australia; Central Clinical School, Monash University, Melbourne, Victoria, Australia; Centre for Epidemiology and Biostatistics, Melbourne School of Population and Global Health, University of Melbourne, Melbourne, Victoria, Australia; Melbourne Sexual Health Centre, Alfred Health, Carlton, Victoria, Australia; Central Clinical School, Monash University, Melbourne, Victoria, Australia; Centre for Epidemiology and Biostatistics, Melbourne School of Population and Global Health, University of Melbourne, Melbourne, Victoria, Australia

**Keywords:** antibacterial agents, fluroquinolones, *Mycoplasma genitalium*, sexually transmitted infections, sitafloxacin

## Abstract

Antimicrobial resistance in *Mycoplasma genitalium* is rising globally and antimicrobial options are limited. We evaluated the efficacy of sitafloxacin regimens for macrolide-resistant *M genitalium* at Melbourne Sexual Health Centre, Australia, between January 2017 and February 2022. Before June 2017, patients received doxycycline followed by sitafloxacin; subsequently, patients received doxycycline followed by combined doxycycline + sitafloxacin. Of 229 patients treated with a sitafloxacin regimen, 80.6% experienced microbial cure. Sitafloxacin cured 94.2% of infections that had not previously failed moxifloxacin and 69.5% of infections that had; prior failure of moxifloxacin was associated with an 8-fold odds of sitafloxacin failure. There was no difference in cure between sequential monotherapy and combination therapy when patients were stratified by past failure of moxifloxacin (*P* > .05); however, small numbers limited comparisons. Sitafloxacin was well tolerated and still achieved 70% cure in patients in whom moxifloxacin had failed. These data highlight the benefit of incorporating relevant fluoroquinolone resistance markers into assays to assist clinical decision making.

## Teaser

Treatment options for macrolide-resistant *Mycoplasma genitalium* are limited and a relatively novel fluoroquinolone, sitafloxacin, has been shown to have 80.6% efficacy.

*Mycoplasma genitalium* is a sexually transmitted bacterium with an evolving antibiotic resistance profile that impacts our ability to cure infections [1]. *Mycoplasma genitalium* has no cell wall, restricting antibiotic choice to agents that act on protein or DNA synthesis including tetracyclines, streptogramins, macrolides, and later-generation fluoroquinolones. The prevalence of *M genitalium* in the general population is estimated to be 1%–3%, and macrolide resistance is reported to be >50% in many settings, including Australia [2]. Extended-spectrum fluoroquinolones, such as moxifloxacin, are used to treat macrolide-resistant *M genitalium*. However, some single-nucleotide polymorphisms (SNPs) in the *parC* gene, such as that conferring the amino acid change S83I, have been shown to increase the minimum inhibitory concentration (MIC) and reduce the efficacy of moxifloxacin [3]. We have shown that these SNPs frequently coexist with macrolide resistance mutations, further restricting treatment options [2].

At Melbourne Sexual Health Centre (MSHC), S83I was detected in 23% of macrolide-resistant strains in 2020 [4], and dual-class resistant infection (macrolide and quinolone resistance) increased from 9% in 2012–2013 to 16% in 2016–2018 [4]. This rise has significantly impacted the efficacy of moxifloxacin in our service with increasing numbers of patients requiring additional antibiotics. We previously reported a small case series of 12 patients with persistent *M genitalium* who had failed moxifloxacin, pristinamycin, and minocycline, and found that treatment with combination 100 mg doxycycline and 100 mg sitafloxacin twice daily (BID) for 1 week cured 11 of 12 infections [5]. Sitafloxacin is a quinolone that is available across the Asia-Pacific region but is limited elsewhere. In Japan, sitafloxacin has been used for a number of years as monotherapy for *M genitalium* and was reported to achieve cure of approximately 90% [6, 7]. However, in Australia, due to concerns about antimicrobial stewardship, sitafloxacin is now reserved for the treatment of resistant *M genitalium* infections. As sitafloxacin is not registered with the Therapeutic Goods Administration in Australia, it is imported from overseas, making it costly and hence reserved for treating resistant *M genitalium* infection. To attain greater understanding of the efficacy and use of sitafloxacin in a larger number of patients, and in the context of rising fluoroquinolone resistance in our clinic population, we undertook an evaluation of all cases treated with sitafloxacin over the past 5 years at MSHC.

## METHODS

We conducted a retrospective evaluation of patients infected with *M genitalium* who were treated with a sitafloxacin regimen at MSHC in Melbourne, Australia, from January 2017 to February 2022. MSHC is the only public clinic treating sexually transmitted infections (STIs) in a city of >5 million. From January 2017 to March 2021, *M genitalium* was tested for using the ResistancePlus MG assay (SpeeDx Pty Ltd, Sydney, Australia). After March 2021, in the final year of the study, the diagnostic assay changed to transcription-mediated amplification (TMA; Aptima MG assay; Hologic Gen-Probe Panther system), with reflex macrolide resistance testing of positives using the ResistancePlus MG assay.

In the first 5 months, from January to May 2017, sitafloxacin was firstline for patients with macrolide-resistant *M genitalium* and prescribed as sequential monotherapy, which involved doxycycline (100 mg BID) for 7 days, followed by sitafloxacin (100 mg BID) for 7 days. Due to antimicrobial stewardship concerns, from June 2017, sitafloxacin was reserved for macrolide-resistant infections that had failed 1 or more other regimens and was used predominantly as sequential combination therapy [8]: doxycycline (100 mg BID) for 7 days followed immediately by combination therapy with doxycycline and sitafloxacin both 100 mg BID for a further 7 days. All patients were asked to return for a test of cure (TOC) 14–21 days after completing sitafloxacin. Clinicians performing a TOC recorded information into an electronic template that captured evolution of genital symptoms after treatment, medication adherence, and side effects, as well as sex since treatment and partner testing and treatment status, to ascertain the risk of reinfection.

Patients were included in the study if they had a TOC performed within 14–90 days of completing sitafloxacin. Patients were excluded if they took less than half of the sitafloxacin doses or had a high risk of reinfection. Consistent with prior studies [4, 8, 9], high risk of reinfection was defined as self-report of condomless sexual intercourse after treatment with a regular partner who had not been tested and/or treated for *M genitalium*. Finally, only a patient's first sitafloxacin treatment event during the study period was included in analyses, and subsequent sitafloxacin treatment events were excluded.

### Data Analysis

Microbial cure was defined as a negative TOC 14–90 days after completing sitafloxacin. Proportions and 95% confidence intervals (CIs) were calculated by exact methods. Logistic regression models with generalized estimating equations were used to explore patient characteristics associated with treatment failure, accounting for multiple diagnoses from individuals who had *M genitalium* detected at >1 anatomical site. We assumed an exchangeable correlation structure and used a cluster-based variance estimate for standard error. Known confounders and characteristics with a significance level of *P* < .05 in univariable analyses were included in the multivariable model, with the following exceptions. Prior treatment failure was reported using 2 different, but correlated measures: (1) number of prior failed antibiotic regimens and (2) prior failure of moxifloxacin. Due to the correlation between the 2 measures, prior treatment failure of moxifloxacin was the variable included in the multivariable model as it had the strongest association with, and greatest clinical relevance to, sitafloxacin failure. We specifically examined correlation and multicollinearity (using variance inflation factors [VIFs]) between independent variables to be included in the multivariable model, and a VIF value ≥2.5 was considered to be an indicator of multicollinearity. When 2 or more variables were deemed to be correlated/colinear, the more clinically relevant variable was included, as described in the Results. We did not include transgender and people who identified as another gender in the logistic regression analyses to avoid bias as there were <5 observations for this variable.

Finally, to evaluate the efficacy of the 2 sitafloxacin regimens used during the study period (ie, sequential monotherapy vs sequential combination therapy), while controlling for prior failure of moxifloxacin, we stratified our study population by prior failure of moxifloxacin versus no prior failure of moxifloxacin. We then assessed the relationship between sitafloxacin regimen and cure within each strata using the Fisher exact test. Stata software (version 17, StataCorp LLC, College Station, Texas) was used for statistical analysis. Ethics approval was obtained from the Alfred Health Ethics Committee (approval number 232/16).

## RESULTS

### Characteristics of the Study Population

**Figure 1.**
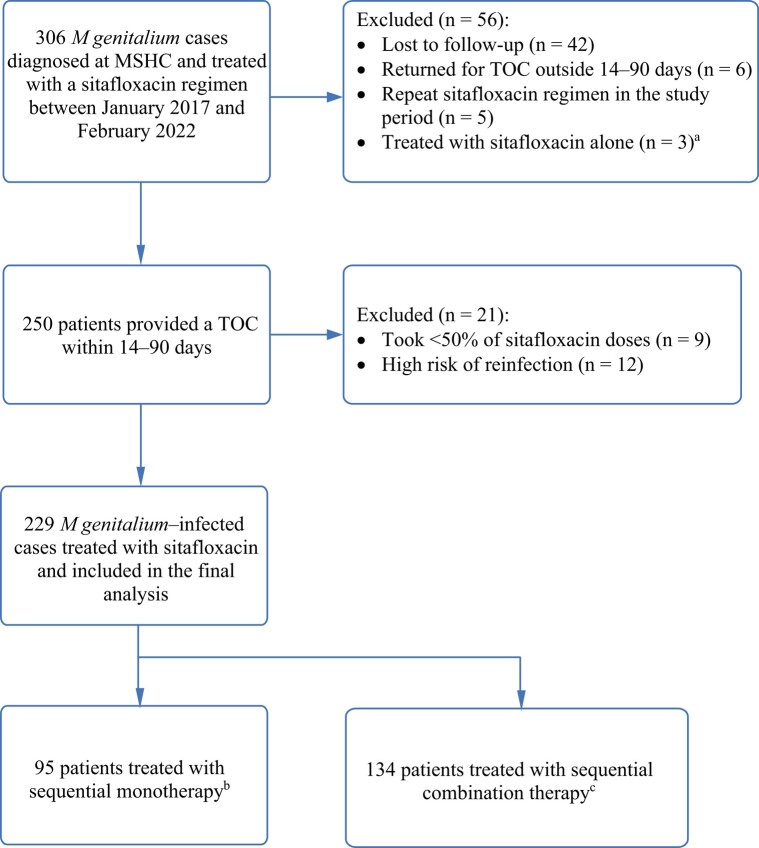
*Mycoplasma genitalium*–infected cases at Melbourne Sexual Health Centre, January 2017–February 2022. ^a^Three patients received sitafloxacin monotherapy 100 mg daily as doxycycline was contraindicated for them. ^b^Sequential monotherapy consisted of doxycycline 100 mg twice daily for 7 days followed by sitafloxacin 100 mg daily for 7 days. ^c^Sequential combination therapy consisted of doxycycline 100 mg twice daily for 2 weeks, with the commencement of sitafloxacin 100 mg daily in the second week of treatment. Abbreviations: MSHC, Melbourne Sexual Health Centre; TOC, test of cure.

Between 3 January 2017 and 14 February 2022, 306 patients were diagnosed with macrolide-resistant *M genitalium* at MSHC and treated with a sitafloxacin regimen (Figure 1). Three patients received sitafloxacin monotherapy due to contraindications to doxycycline and were excluded. Of the remaining 303 patients, 250 patients (82.5%) had a TOC recorded within 14–90 days. Twenty-one of 250 cases were excluded: 9 took <50% of the prescribed sitafloxacin doses and 12 cases had a high risk of reinfection. As a result, 229 patients were included in the analysis. A total of 95 of 229 patients received sequential doxycycline followed by sitafloxacin (each drug for 7 days) and 134 received doxycycline followed by sitafloxacin with doxycycline (doxycycline for 14 days, with sitafloxacin for the last 7 days) (Figure 1).

Of the 229 cases, 96 (41.9%) were men who have sex with men, 63 (27.5%) were heterosexual men, 67 (29.3%) were female, and 3 (1.3%) were transgender or identified as another gender (Table 1). The median age of participants was 29 years (interquartile range, 25–34 years). Three of the 229 patients had a multisite infection, representing 232 sites of infection. There were 126 (54.3%) male (at birth) urethral infections, 69 (29.7%) cervicovaginal infections (53 detected on a cervicovaginal swab and 16 detected in a first-pass urine sample), and 37 (16.0%) anorectal infections. Of the 3 patients with multisite infections, 2 had both urine and anorectal infections, and 1 had an anorectal and cervicovaginal infection.

**Table 1.** Characteristics of 229 Patients Who Received a Sitafloxacin Regimen Between January 2017 and February 2022

Characteristic	Total (N = 229)
Age, y, median (IQR)	29 (25–34)
Gender/sexual orientation	
Female	67 (29.3)
Heterosexual male	63 (27.5)
MSM	96 (41.9)^a^
Transgender/other	3 (1.3)^b^
Sample, site of infection (n = 232)	
Urethral	126 (54.3)
Anorectal swab	37 (16.0)
Cervicovaginal sample^c^	69 (29.7)
Indication for test	
Persistent asymptomatic *M genitalium*	156 (67.2)
Male urethritis	44 (19.0)
Rectal symptoms	9 (3.9)
Female genital/pelvic symptoms	8 (3.4)^d^
Other symptoms	3 (1.3)
Asymptomatic contact	12 (5.2)
Failure of prior antibiotic regimens	
None	94 (41.1)
Failure of 1 prior regimen	70 (30.6)
Failure of ≥2 prior regimens	65 (28.4)
Prior failure of moxifloxacin	
No	101 (44.1)
Yes	128 (55.9)
Adherence	
No missed doses documented	203 (87.5)
Missed 1–6 doses	13 (5.6)
Unknown	13 (5.6)
Adverse effects	
Nausea	18 (7.9)
Diarrhea	27 (11.8)
Abdominal pain	10 (4.4)
Headache	3 (1.3)
Dizziness	4 (1.7)
Fatigue/lethargy	4 (1.7)
Tendon pain	5 (2.2)
Other	27 (11.8)
Not recorded	18 (7.9)
None	131 (57.2)

Data are presented as No. (%) unless otherwise indicated.

Abbreviations: IQR, interquartile range; MSM, men who have sex with men.

^a^Two had multisite infections (urethral and anorectal).

^b^One had multisite infections (cervicovaginal and anorectal).

^c^This comprised 53 cervicovaginal swabs and 16 first-pass urine samples.

^d^Urinary symptoms (n = 1), abnormal bleeding (n = 3), pelvic pain (n = 3), abnormal vaginal discharge (n = 1).

The most common indication for testing was urethritis in men (n = 44 [19.0%]); however, most cases were asymptomatic by the time they received a sitafloxacin regimen (n = 168 [73.4%]). Screening for asymptomatic *M genitalium* is not practiced or recommended at MSHC. The asymptomatic patients represent those who had initially been symptomatic but had failed other regimens and had persistent asymptomatic infection (n = 156), and a small number (n = 12) who were asymptomatic contacts of patients with resistant infection.

### Previous Antibiotic Therapy

Of the 229 patients, 94 (41.1%) had not received prior treatment for their infection (representing patients in the first 5 months of the study period), 70 (30.6%) had failed 1 prior antibiotic regimen, and 65 (28.4%) had failed at least 2 prior regimens. During the first 5 months of the study period, 6% (n = 6/93) of patients had failed ≥1 prior regimen. From June 2017, 93% (n = 123/142) had failed ≥1 prior regimen. Of note, 128 (55.9%) patients had previously received and failed moxifloxacin. Additional antibiotics that patients had been exposed to included azithromycin (n = 25), pristinamycin (n = 30), and minocycline (n = 30).

### Adherence and Adverse Effects

Adherence data were available for 216 (94.3%) cases. Most patients (n = 203 [94.0%]) reported not missing any doses and 13 (6.0%) reported missing between 1 and 6 doses. Tolerability data were available for 211 cases (92.1%); 80 (37.9%) patients reported side effects. Side effects were mild and self-limiting, and no patients ceased treatment early because of side effects. The most common side effects reported were diarrhea (n = 27), nausea (n = 18), and abdominal pain (n = 10).

### Microbiological Cure and Factors Associated With Sitafloxacin Failure

Of 232 infections, 187 (80.6% [95% CI, 74.9%–85.5%]) experienced microbial cure following sitafloxacin and 45 (19.4% [95% CI, 14.5%–25.1%]) failed sitafloxacin. Of the 3 patients with multisite infections, 2 had infections in the urethral and anorectum, both of which were cured at both sites with sitafloxacin; 1 patient had cervicovaginal and anorectal infections and was cured only at the anorectal site, giving 1 discordant result.

In univariable analyses, being female or (by anatomical site) having a cervicovaginal infection was significantly associated with sitafloxacin failure (odds ratio [OR], 3.35 [95% CI, 1.48–7.58], *P* = .004 and OR, 2.48 [95% CI, 1.24–4.98], *P* = .011, respectively; Table 2). Infections that had previously been treated with 1 or >1 prior antibiotic regimen were more likely to fail sitafloxacin compared to treatment-naive infections (OR, 4.31 [95% CI, 1.71–10.82], *P* = .002 and OR, 5.53 [95% CI, 2.21–13.81], *P* < .001, respectively). Unsurprisingly, prior failure of moxifloxacin was very strongly associated with an increased odds of sitafloxacin failure (OR, 6.83 [95% CI, 2.83–16.51], *P* < .001). Use of the Aptima TMA MG assay was also significantly associated with sitafloxacin failure (OR, 3.11 [95% CI, 1.59–6.07], *P* = .001) by univariable analysis. However, this was likely a spurious association, as at the time this assay was used, patients who received sitafloxacin had previously failed prior treatment regimens, predominately reflecting prior failure of moxifloxacin (ie, moderate correlation was observed between the use of the *M genitalium* TMA assay and prior failure of moxifloxacin; ρ = 0.498, *P* < .001). Finally, infections treated with sitafloxacin as sequential combination therapy were more likely to fail treatment compared to infections treated with sequential monotherapy (26.9% vs 9.2%; OR, 3.56 [95% CI, 1.64–7.72], *P* = .001), which was again likely due to the fact that more heavily pretreated patients received sequential combination therapy. Age and treatment adherence were not associated with sitafloxacin failure.

**Table 2.** Characteristics Associated With Failure of Sitafloxacin in Patients With *Mycoplasma genitalium* Infection^a^

Characteristic	Cured, No. (% [95% CI])	Failed, No. (% [95% CI])	Unadjusted OR (95% CI)	*P* Value	Adjusted OR (95% CI)	*P* Value
Total infections (n = 232)	187 (80.6 [74.9–85.5])	45 (19.4 [14.5–25.1])	99.9 (.95–1.05)	.975	…	
Age, y, median (IQR)	29 (25–33)	29 (25–34)	…		1.03 (.97–1.08)	.361
Gender/orientation						
MSM	87 (46.5 [39.2–53.9])	11 (24.4 [12.9–39.5])	1		1	
Female	47 (25.1 [19.1–32.0])	20 (44.4 [29.6–60.0])	3.35 (1.48–7.58)	.004	1.89 (.75–4.78)	.178
Heterosexual male	52 (27.8 [21.5–34.8])	11 (24.4 [12.9–39.5])	1.66 (.67–4.11)	.269	0.99 (.37–2.60)	.979
Transgender/other^b^	1 (0.5 [.00–2.9])	3 (6.7 [1.4–18.3])	…		…	
Site of infection^c^						
Urethral	106 (56.7 [49.3–63.9])	20 (44.4 [29.6–60.0])	1		…	
Anorectal	34 (18.2 [12.9–24.5])	3 (6.7 [1.4–18.3])	0.47 (.13–1.68)	.244	…	
Cervicovaginal	47 (25.1 [19.1–32.0])	22 (48.9 [33.7–64.2])	2.48 (1.24–4.98)	.011	…	
Failure of prior antibiotic regimens						
No	90 (48.1 [40.8–55.5])	7 (15.6 [6.5–29.5])	1		…	
Failure of 1 prior regimen	52 (27.8 [21.5–34.8])	18 (40.0 [25.7–55.7])	4.31 (1.71–10.82)	.002	…	
Failure of ≥2 prior regimens	45 (24.1 [14.1–30.8])	20 (44.4 [29.6–60.0])	5.53 (2.21–13.81)	<.001	…	
Prior failure of moxifloxacin						
No	98 (52.4 [45.0–59.7])	6 (13.3 [5.1–26.8])	1		1	
Yes	89 (47.6 [40.3–55.0])	39 (86.7 [73.2–94.9])	6.83 (2.83–16.51)	<.001	7.56 (2.38–24.04)	<.001
Sitafloxacin regimen						
Sequential monotherapy	89 (47.6 [40.3–55.0])	9 (20.0 [9.5–34.6])	1		…	
Sequential combination therapy	98 (52.4 [45.0–59.7])	36 (80.0 [65.4–90.4])	3.56 (1.64–7.72)	.001	…	
Adherence^d^						
No missed doses	161 (92.5 [80.3–90.7])	45 (100.0 [92.1–100])	…		…	
Missed 1–6 doses	13 (7.5 [2.8–11.6])	0 (0.0 [.0–7.9])	Omitted	.075^e^	…	
*Mycoplasma genitalium* assay used^f^						
ResistancePlus MG assay	140 (74.9 [68.0–80.9])	22 (48.9 [33.7–64.2])	1		1	
Aptima MG assay	47 (25.1 [19.1–32.0])	23 (51.1 [35.8–66.3])	3.11 (1.59–6.07)	.001	1.46 (.68–3.12)	.328

Abbreviations: CI, confidence interval; IQR, interquartile range; MG, *Mycoplasma genitalium*; MSM, men who have sex with men; OR, odds ratio.

^a^Two hundred twenty-nine patients infected with MG representing 232 anatomical sites of infection.

^b^We did not include transgender people and people who identified as another gender in logistic regression analyses to avoid bias as there were <10 observations.

^c^First-pass urine samples were obtained from people who were male at birth. Of the 69 cervicovaginal infections, 53 were detected with cervicovaginal swabs and 16 were detected in first-pass urine samples.

^d^Adherence data available for 216 patients (219 infections).

^e^Calculated using 1-sided Fisher exact test.

^f^Prior to March 2021, the ResistancePlus MG assay (SpeeDx) was used for *M genitalium* testing; after March 2021, transcription-mediated amplification (Aptima MG assay; Hologic Gen-Probe Panther system) was used.

We included age, gender, prior failure of moxifloxacin, and *M genitalium* assay in a multivariable model (Table 2). Type of sitafloxacin regimen (ie, sequential monotherapy vs sequential combination therapy) was not included in the model due to high correlation and collinearity with prior failure of moxifloxacin (ρ = −0.844, *P* < .001), and the latter was selected for inclusion in the model due to its clinical relevance to treatment outcome. As stated, the *M genitalium* assay used (ie, ResistancePlus MG assay vs Aptima TMA MG assay) was also moderately correlated with prior failure of moxifloxacin (ρ = 0.498, *P* < .001); however, it was able to be included in the multivariable model as there was no evidence of multicollinearity (VIF <2.5). Site of infection was not included due to its correlation with gender, and number of prior failed antibiotic regimens was not included due to its correlation with prior failure of moxifloxacin. Overall, in the adjusted analysis, prior failure of moxifloxacin was the only characteristic that remained highly significantly associated with increased odds of sitafloxacin failure (adjusted OR [AOR], 7.56 [95% CI, 2.38–24.04], *P* < .001).

Due to the high correlation and collinearity between prior failure of moxifloxacin and type of sitafloxacin regimen, we stratified the study population by prior failure of moxifloxacin (Table 3) and assessed the relationship between type of sitafloxacin regimen and cure within each strata, acknowledging that numbers were small in some groups. Overall, among people who had not previously failed moxifloxacin, 98 of 104 (94.2% [95% CI, 87.9%–97.9%]) infections were cured by sitafloxacin: 11 of 12 (91.7% [95% CI, 61.5%–99.8%]) given sequential combination therapy were cured compared to 87 of 92 (94.6% [95% CI, 87.8%–98.2%]) infections following sequential monotherapy (*P* = .530), with no significant difference between groups. Among people who had previously failed moxifloxacin, 89 of 128 (69.5% [95% CI, 60.8%–77.4%]) infections were cured by sitafloxacin: 87 of 122 (71.3% [95% CI, 62.4%–79.1%]) infections given sequential combination therapy were cured compared to 2 of 6 (33.3% [95% CI, 4.3%–77.7%]) infections given sequential monotherapy (*P* = .069). While there was an obvious difference between these estimates, small numbers heavily impacted this comparison.

**Table 3.** Microbial Cure Following Sequential Monotherapy Versus Sequential Combination Therapy Stratified by Prior Failure of Moxifloxacin

Type of Therapy	Prior Moxifloxacin Failure (n = 128)		No Prior Moxifloxacin Failure (n = 104)	
	Cured	*P* Value^a^	Cured	*P* Value^a^
Sequential monotherapy (n = 98)	2/6 (33.3%)	.069	87/92 (94.6%)	.530
Sequential combination therapy (n = 134)	87/122 (71.3%)		11/12 (91.7%)	
Total	89/128 (69.5%)		98/104 (94.2%)	

^a^Calculated using Fisher exact test.

## DISCUSSION

This study reports the efficacy of sitafloxacin for *M genitalium* infections in the setting of rising macrolide and fluoroquinolone resistance and increasingly limited treatment options. The overall cure rate of a sitafloxacin regimen was 80% in our population, with an 8-fold increased odds of failing sitafloxacin in patients who had previously failed moxifloxacin, likely reflecting the presence of existing fluoroquinolone resistance mutations in the *parC* and/or *gyrA* genes. Unfortunately, sequencing of these samples was not available due to the impact of coronavirus disease 2019 (COVID-19) on our laboratory services over the study period, but we know that the prevalence of clinically relevant *parC* mutations exceeded 20% in *M genitalium* infections in our clinic population during this study [4, 10, 11]. In addition, it is anticipated that approximately 30% of *M genitalium* infections with a *parC* mutation will have a concurrent *gyrA* mutation, further impacting on treatment outcome [7, 10]. Sitafloxacin cured 94% of *M genitalium* infections that had not previously failed moxifloxacin compared to 69% of infections that had, highlighting the value of this immediately available information to clinicians to inform of decision making. This could also suggest a lower prevalence of *parC* and/or *gyrA* mutations in the first 5 months of the study, in keeping with data that show this prevalence has slowly increased in our population [10]. Our series was unable to establish if sequential combination therapy was superior to sitafloxacin sequential monotherapy due to changes in the indications for sitafloxacin use and a number of highly correlated variables. In particular, prior failure of moxifloxacin was far more common in patients given sequential combination therapy compared to sequential monotherapy. While our stratified analysis was impacted by small numbers, it did suggest that there was likely to be limited or no benefit of sequential combination therapy over sequential monotherapy in patients who had not failed moxifloxacin. In patients who had failed moxifloxacin, it is possible that sequential combination therapy offers some benefit over sequential monotherapy, but larger sample sizes and accompanying *parC/gyrA* sequencing data are needed to assess this.

Sitafloxacin, a relatively novel fluoroquinolone with bactericidal effects, exhibits in vitro antibacterial activity against gram-positive, gram-negative, and anaerobic bacteria, as well as atypical pathogens [12]. It has been shown to have potent activity against multidrug-resistant organisms, displaying lower MICs and lower rates of resistance compared to ciprofloxacin and levofloxacin [13]. It has been used to treat respiratory and urinary tract infections in Japan for decades, and became available in Thailand and other parts of the Asia-Pacific in the past decade [14, 15]. Sitafloxacin is well tolerated, with the most common adverse effect being gastrointestinal disturbance [12].

In adjusted analysis, patients who had previously failed treatment with moxifloxacin were almost 8 times more likely to fail treatment with sitafloxacin compared to those who had not previously failed moxifloxacin, although sitafloxacin still cured 70% of these infections. In the absence of sequencing data, this is likely to be due to specific mutations in the *parC* gene including the most common *parC* SNP, G248T, which confers the amino acid change S83I [11, 16]. In vitro, sitafloxacin exhibits lower MICs in isolates with *parC* S831 mutations than moxifloxacin [3]. Over the past decade at our clinic, there has been a concerning rise in the proportion of *M genitalium* infections with *par*C S83I mutations from 13.0% in 2012–2013 to 27.5% in 2019–2020 [10]. Importantly, a significant proportion of infections with an S83I mutation also had a concurrent *gyrA* gene mutation (ie, G285A/M95I), and this has increased from 28.2% in 2016–2018 to 34.8% in 2019–2022 [10]. The addition of this *gyrA* gene mutation to the *parC* S83I mutation significantly reduces the efficacy of both moxifloxacin and sitafloxacin [10]. These data highlight the importance of understanding the prevalence of key fluoroquinolone resistance markers in populations and the increasing value of assays that include relevant SNPs in both genes [4, 17] to guide therapeutic decision making for *M genitalium*, particularly in settings where fluoroquinolone resistance is common. A systematic review and meta-analysis of studies published up to 2019 reported the summary prevalence estimate of mutations associated with fluoroquinolone resistance was 8% worldwide, with the highest prevalence of these mutations in the Western Pacific Region [2]. Notably, in Japan there was a significant increase in fluoroquinolone resistance–associated mutations from 5% before 2010 to 29% in 2016–2017 [18], which is similar to what has been observed in our clinic population [10].

Preliminary in vitro data have shown a synergistic effect from the combination of doxycycline and sitafloxacin in *M genitalium* strains without *parC* mutations; however, this has not been evaluated for highly resistant strains (J. S. Jensen, personal communication). After our initial small series demonstrating high cure in patients with no further treatment options [5], we hoped to determine if sitafloxacin in combination with doxycycline was superior to sitafloxacin as monotherapy. However, due to the changes in indications for sitafloxacin use and correlation between sequential combination therapy and past failure of moxifloxacin, it was not possible to assess this in regression analyses. While we conducted a stratified analysis, this was significantly impacted by small numbers. Stratified data did suggest that overall cure was high (>90%) in patients who had not previously failed moxifloxacin, regardless of whether sitafloxacin was used as monotherapy or in combination with doxycycline. In patients who had previously failed moxifloxacin, sequential monotherapy cured 33% of infections, while sequential combination therapy cured 71%, suggesting there may be a benefit of sequential combination therapy in heavily pretreated patients. However, as mentioned, small numbers impacted this comparison, and larger sample sizes with sequencing data are needed to determine if sequential combination therapy offers any benefit over sequential monotherapy in heavily pretreated patients with existing resistance.

This study has strengths and limitations. MSHC is the largest STI service in Australia, with 60 000 consultations per year, and services a diverse population of >5 million people. All patients who present for a TOC at MSHC see a clinician who uses a template to gather important clinical information, including ongoing symptoms, adherence, and side effects to medications, as well as risk of reinfection. Therefore, these data were collected in a standardized format for all patients. However, these data are self-reported and therefore prone to recall and reporting bias. Additionally, MSHC is a specialist sexual health clinic and findings from this service may not be generalizable to the wider community. Patients who present to sexual health clinics tend to have had higher exposure to prior antibiotics, further increasing the chance of resistance. As mentioned above, during the first 5 months of the study period, sitafloxacin was primarily used firstline for macrolide-resistant *M genitalium* as sequential monotherapy. In the latter part of the study period, sitafloxacin was used as sequential combination therapy and primarily used for patients who had failed ≥1 prior regimen. As a consequence, a number of variables examined in univariable analyses, including the type of sitafloxacin regimen and failure of prior regimens, were highly correlated and this limited our ability to compare the efficacy of the 2 different sitafloxacin regimens. Furthermore, 2 different *M genitalium* diagnostic assays were used during the study period, which may have influenced our cure estimates; however, in adjusted analyses, there was no significant difference in the detection of *M genitalium* between the 2 assays. Finally, the COVID-19 pandemic impacted our service; testing laboratories changed 3 times in this period and we were unable to obtain access to all samples for sequencing of resistance-associated genes.

In conclusion, in a clinic population where the prevalence of clinically relevant *parC* mutations exceeds 20%, we found that sitafloxacin was well tolerated and cured 81% of macrolide-resistant *M genitalium* infections, many of which had failed several other regimens. There was a higher likelihood of sitafloxacin failure in patients who previously failed moxifloxacin, but cure still occurred in 70% of these patients, which is important information for clinicians managing patients with limited treatment options. In the context of rising resistance in *M genitalium*, these data indicate benefit in knowing the prevalence and trends in fluoroquinolone resistance markers in clinic populations and value in incorporating them into assays to help with clinical decision making.

## Notes

***Acknowledgments*.** The authors do not have an association that might pose a conflict of interest.

***Patient consent*.** This study does not include factors necessitating patient consent.

***Financial support.*** C. K. F. and C. S. B. are supported by Australian National Health and Medical Research Council (NHMRC) Leadership Investigator Grants (grant numbers GNT1172900 and GNT1173361, respectively) and E. P. F. C. by an NHMRC Emerging Leadership Investigator Grant (grant number 1172873).
